# 1666. A Clinical Care Pathway Improves Antibiotic Prescribing in Hospitalized Children with Community Acquired Pneumonia

**DOI:** 10.1093/ofid/ofad500.1499

**Published:** 2023-11-27

**Authors:** Jennifer Schweiger, Nicole M Poole, Jillian Cotter, Lilliam Ambroggio, Christine MacBrayne

**Affiliations:** Atrium Health, Denver, Colorado; University of Colorado School of Medicine, Aurora, Colorado; University of Colorado, Aurora, Colorado; Children's Hospital Colorado, Aurora, Colorado; Children's Hospital Colorado, Aurora, Colorado

## Abstract

**Background:**

Amoxicillin for a 5-day duration is the first-line recommended treatment for pediatric uncomplicated community acquired pneumonia (CAP). This project sought to identify the impact of a revised AgileMD clinical care pathway (CCP) on antibiotic prescribing and clinical outcomes.

**Methods:**

In April 2021, a revised CAP CPP was implemented into clinical practice. Changes included prioritizing amoxicillin as the treatment of choice, discouraging use of broad-spectrum antibiotics and azithromycin, and decreasing treatment duration to 5 days. In this quasi-experimental study patients, 60 days-18 years, admitted from January 2018 to July 2022 who were diagnosed and treated with antibiotics for uncomplicated CAP were included. Patients with complex chronic conditions, respiratory failure at presentation, septic shock, complicated CAP, and hospitalization in the prior 30 days were excluded. Data collected included patient demographics, admission diagnoses, antibiotic prescribing, and 7-day readmission rates. Primary outcomes included (1) percent of patients receiving each antibiotic and (2) median days of total antibiotic therapy (inpatient plus outpatient) for CAP. Statistical process control charts, Fisher exact and Mann Whitney U tests were utilized to compare clinical characteristics and antibiotic prescribing practices between the pre-CCP (1/2018-3/2021) and post-CCP (4/2021-7/2022) groups.

**Results:**

Of 900 CAP encounters that met inclusion criteria, 65% were pre-intervention period and 35% post-intervention (Table 1). Most patients received supplemental oxygen at presentation (N=819; 91%) and nearly all patients had a chest x-ray obtained (N=890; 97%). Prescribing of amoxicillin increased from 67% to 78%. The largest decrease occurred in azithromycin prescribing from 24% to 3% (Figure 1). Duration of therapy in the post-CCP group was statistically shorter compared to the pre-CCP group (7.5 days vs 5.9 days) (Figure 2). Readmission rates were not statistically different between groups (p=0.33).

**Table 1.**
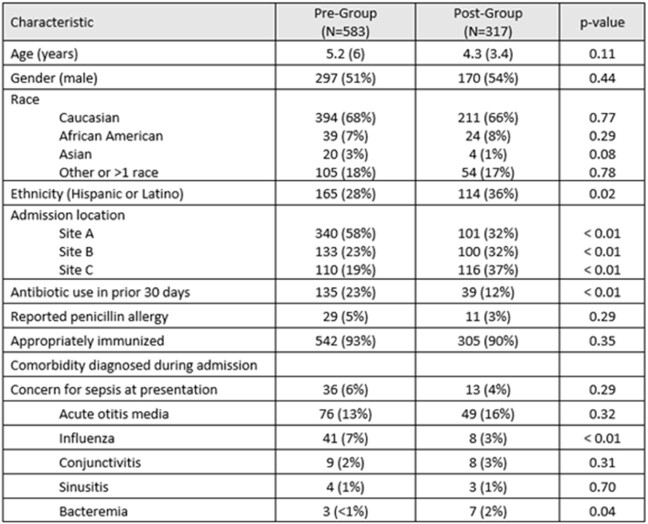
Characteristics of Patients Hospitalized with CAP in Pre- and Post-Intervention Groups Data are median (IQR) or count (%); p-values calculated with Fisher's exact or Mann-Whitney U tests.

**Figure 1.**
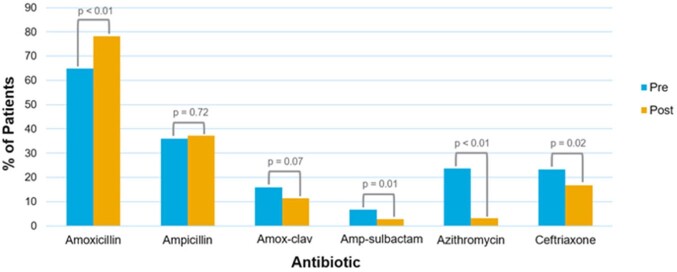
Percentage of Patients Treated with the Six Most Common Antibiotic Choices in Pre- and Post-Intervention Groups Data are percent of patients per time group; p-values calculated with Fisher’s exact test. Data for clindamycin, levofloxacin, vancomycin, cefdinir, cefpodoxime, cefixime, clarithromycin are not shown.

**Conclusion:**

Clinical pathway incorporation into the EMR significantly improved antibiotic choice and decreased duration for children with CAP.

**Figure 2.**
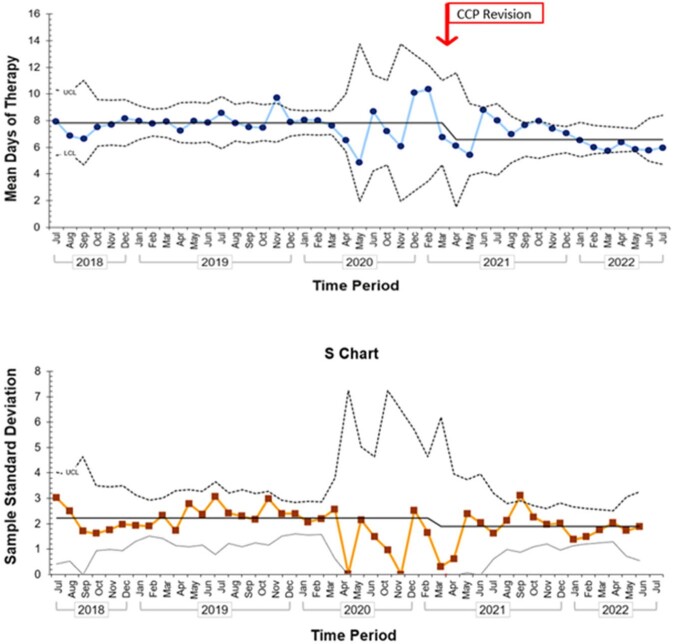
Mean Days of Therapy Prescribed By Month for Patients Admitted with CAP Before and After CCP Revision Mean days of therapy are represented with an X-bar and S control chart.

**Disclosures:**

**Jillian Cotter, MD, MSCS**, Pfizer: Grant/Research Support **Lilliam Ambroggio, PhD**, Pfizer Inc.: Grant/Research Support

